# A Man with Unilateral Endophthalmitis: A Case of Disseminated Nocardiosis

**DOI:** 10.1155/2015/607421

**Published:** 2015-03-23

**Authors:** N. Navarrete-Navarrete, J. Escobar Sevilla, M. Toribio García, F. Urbano, J. M. Sabio, J. Jiménez-Alonso

**Affiliations:** ^1^Unit of Autoimmune Diseases, Service of Internal Medicine, University Hospital Virgen de las Nieves, Avenida Fuerzas Armadas 2, 18014 Granada, Spain; ^2^Service of Internal Medicine, University Hospital Virgen de las Nieves, Avenida Fuerzas Armadas 2, 18014 Granada, Spain; ^3^Service of Ophthalmology, University Hospital Virgen de las Nieves, Avenida Fuerzas Armadas 2, 18014 Granada, Spain

## Abstract

We present the case of a patient with an infection by Nocardia which manifested itself with monocular endophthalmitis. Nocardia infection is not common and ocular involvement is one of the most uncommon presentations. In these cases it is very important to make an early diagnosis and intensive treatment to prevent the visual prognosis.

## 1. Introduction

Painful red eye is one of the diseases most common in ophthalmology services. Its etiological study should consider infectious, inflammatory, and paraneoplasic causes, although in a nonnegligible number of cases the cause is unknown. We present a patient consulting by painful red eye, as a rare presentation of an uncommon systemic infection.

## 2. Case History

A 60-year-old male with no relevant past medical history complained of intense pain, reduced visual acuity, and reddening of the left eye that had lasted a week and had not improved despite treatment with topical anti-inflammatories. He was examined by an ophthalmologist and diagnosed with acute unilateral panuveitis in the left eye and initiated treatment with prednisone 60 mg/day but still observed no improvement.

A dry cough lasting several months, loss of appetite, and weight loss of 2 kg in the previous 2 months stood out in the patient's medical history.

During the ophthalmological examination the Tyndall effect was particularly evident without hypopyon. Conjunctival hyperaemia and rubeosis iridis were observed and IOP was 18 mmHg.

In the general physical examination dispersed rhonchi sounds were detected in both hemithoraces and mild pain during palpation of the right hypochondrium was observed. The liver was not enlarged and there were no other significant findings. The rest of the physical examination was normal.

Ophthalmological progress was slow due to the presence of progressive erythema and orbital swelling of the left eye and an increase in IOP of the left eye. The patient also presented with deteriorated breathing, a persistent dry cough, and breathlessness after minimal exertion. He was administered parenteral antibiotics (ceftriaxone) and treatment for ocular hypertension and was admitted into hospital.

The blood tests were normal and the biochemistry showed CRP 11 mg/dL, and the rest of the parameters were normal. The following tests were normal or negative: serological testing for cytomegalovirus and herpes virus, hepatitis B and C, toxoplasma, HIV, syphilis, and tuberculin skin test.

**Figure 1:**
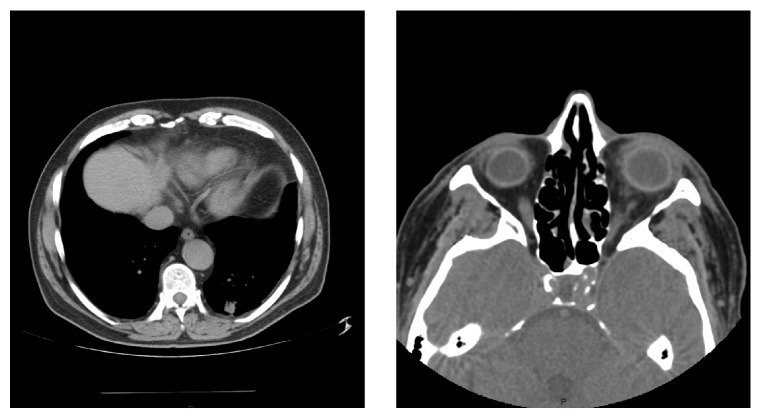
Orbital and lung CT scan.

An abdominal ultrasound showed several small hypodense lesions compatible with cysts and the chest and abdomen CT highlighted irregular, alveolar-shaped pseudonodules measuring 20 × 17 mm and 10 × 19 mm in the left lower lobe (see Figure 1).

A bronchoscopy with bronchoalveolar lavage was performed and there were no microbiological or cytological findings.

Given the absence of an aetiological diagnosis, a vitrectomy was performed and the growth of* Nocardia otitidiscaviarum* resistant to imipenem, tetracyclines, clarithromycin, amoxicillin, and cefotaxime and susceptible to TMP-SMX was detected. Therapy with TMP-SMX was initiated, leading to ophthalmological and respiratory improvement. After 2 months, both the ocular inflammation and the liver and lung lesions had disappeared, as shown by the imaging tests.

## 3. Discussion

*Nocardia* spp. infection is caused by a slow-growing, aerobic, Gram-positive microorganism that is inhaled from organic matter or aerosolized water.

In general, nocardia infections of the eye are very uncommon, especially in nonimmunosuppressed patients [1, 2].

Cases of endophthalmitis described in the literature have been linked to infection after surgery or trauma [3], which was not the case for our patient.

In cases of systemic infection, the mortality rate can be as high as 15% [4], which is why early antibacterial treatment is crucial. Furthermore, treatment should be adjusted to take into account the in vitro sensitivity and resistance profile.

Atypical presentations of nocardia should be considered in patients presenting with endophthalmitis and other systemic symptoms.
